# Introduction to Special Issue “Neurocognitive Processes: Measurement, Connections to Academic Achievement and Clinical Applications”

**DOI:** 10.3390/jintelligence13080097

**Published:** 2025-08-04

**Authors:** George K. Georgiou

**Affiliations:** Department of Educational Psychology, University of Alberta, 3-102A Education North, Edmonton, AB T6G 2G5, Canada; georgiou@ualberta.ca; Tel.: +1-780-492-8247

Several studies have shown that intelligence is a significant correlate of academic achievement. Despite its acknowledged importance, there are a few issues related to intelligence that this Special Issue aimed to address. First, some researchers have argued that the relationship between intelligence and academic achievement may have been confounded by the fact that popular batteries of intelligence include measures of vocabulary and mathematics in the estimation of an IQ score, which are too close to the outcome measures they aim to predict, thus creating a vicious circle. To bypass this problem, Das and colleagues (e.g., Das et al. 1994) proposed measuring intelligence in terms of neurocognitive processes like Planning, Attention, Simultaneous and Successive (PASS) processing. The PASS theory of intelligence was based on Alexander Luria’s influential work on how the brain system is organized and how the different processing units interact with each other to help an individual attain their goals. Although PASS theory of intelligence has been around for more than four decades (see Georgiou et al. 2020, for a meta-analysis), it has attracted less interest from researchers compared to other theories of intelligence and little is known about its many applications in psychology and education. For this reason, in this Special Issue, I aimed to bring some of the work around PASS theory to the forefront.

A second issue that remains unclear regarding PASS theory is the influence of culture and race on these cognitive processes. Naglieri et al. (2005; see also Naglieri and Otero 2024, in this Special Issue) have argued that the Cognitive Assessment System (CAS; the battery of tasks used to operationalize PASS processes) is culturally fair and allows us to measure students’ cognitive processes that are not confounded by their language skills. This is particularly relevant today when we advocate for equity in education. Finally, the clinical applications of using CAS (particularly in identifying interesting profiles of students) remain understudied.

This Special Issue includes six papers: five empirical studies and a review. Two of the studies were conducted in China and one was conducted in Japan, Cyprus, and Puerto Rico, respectively. Two of the studies explored the clinical applications of CAS (see Okazaki et al. 2024; and Georgiou et al. 2024), one was an intervention study (see Cai et al. 2024), one explored the strategies used during the planning tasks in CAS (Cordero-Arroyo et al. 2024) and one evaluated the psychometric properties of a complex problem-solving task with connections to the planning construct in CAS (Li et al. 2024).

It is obvious from these publications that there has been a paradigmatic shift in the research on PASS theory, namely researchers focus more on the clinical applications of the theory or on certain processes such as planning. For example, Georgiou et al. (2024) examined how researchers and practitioners could use CAS-2: Brief and the discrepancy consistency method (DCM) to identify cognitive strengths and weaknesses that are related to academic achievement in an unselected sample of Grade 6 children. Detecting significant variability in PASS scores is particularly important because it has significant instructional implications. This is nicely illustrated in Cai et al.’s (2024) intervention study with Grade 1 to 4 Chinese students with low attention. Using the Children’s Mathematics and Cognition Training program, which consists of five modules (shifting patterns, number line, counting, mapping and estimating, and memory span for numbers), Cai and colleagues showed that they could improve not only cognitive planning and simultaneous processing but also children’s mathematics performance.

A lingering question concerns where we see PASS theory of intelligence go from here. As put by Georgiou and Das (2019) “is it a frozen dinner or a moving feast”? I would like to think of it as a “moving feast”, an alternative view of intelligence that has been dominated by dichotic approaches (e.g., crystallized versus fluid intelligence; verbal versus nonverbal intelligence). This Special Issue has showcased some promising directions in research around PASS theory but has also revealed areas where more research is needed. For example, we need more studies on the developmental relations between PASS processes and academic achievement and more intervention studies using the programs that were developed based on PASS theory.
